# Tunable Raman Gain in Transparent Nanostructured Glass-Ceramic Based on Ba_2_NaNb_5_O_15_ †

**DOI:** 10.3390/nano13071168

**Published:** 2023-03-24

**Authors:** Pasquale Pernice, Luigi Sirleto, Manuela Rossi, Mario Iodice, Alessandro Vergara, Rocco Di Girolamo, Giuseppina Luciani, Claudio Imparato, Antonio Aronne

**Affiliations:** 1 Dipartimento di Ingegneria Chimica, dei Materiali e della Produzione Industriale, Università degli Studi di Napoli Federico II, P.le Tecchio, 80, I-80125 Napoli, Italy; 2 National Research Council (CNR), Institute of Applied Sciences and Intelligent Systems, Via Pietro Castellino 111, I-80131 Naples, Italy; 3 Dipartimento di Scienze della Terra dell’Ambiente e delle Risorse, Università degli Studi di Napoli Federico II, Complesso Universitario di M. S. Angelo, Via Cinthia 21, I-80126 Napoli, Italy; 4 National Research Council (CNR), Institute of Crystallography, Via Amendola 122/o, I-70126 Bari, Italy; 5 Dipartimento di Scienze Chimiche, Università degli Studi di Napoli Federico II, Via Cinthia, I-80126 Napoli, Italy

**Keywords:** transparent glass-ceramics, nonlinear optical properties, crystalline nanostructuring, Raman gain

## Abstract

Stimulated Raman scattering in transparent glass-ceramics (TGCs) based on bulk nucleating phase Ba_2_NaNb_5_O_15_ were investigated with the aim to explore the influence of micro- and nanoscale structural transformations on Raman gain. Nanostructured TGCs were synthesized, starting with 8BaO·15Na_2_O·27Nb_2_O_5_·50SiO_2_ (BaNaNS) glass, by proper nucleation and crystallization heat treatments. TGCs are composed of nanocrystals that are 10–15 nm in size, uniformly distributed in the residual glass matrix, with a crystallinity degree ranging from 30 up to 50% for samples subjected to different heat treatments. A significant Raman gain improvement for both BaNaNS glass and TGCs with respect to SiO_2_ glass is demonstrated, which can be clearly related to the nanostructuring process. These findings show that the nonlinear optical functionalities of TGC materials can be modulated by controlling the structural transformations at the nanoscale rather than microscale.

## 1. Introduction

Nonlinear conversion is a fundamental aspect of nonlinear photonics because it should allow the realization of efficiently integrated coherent light sources. Several second and third order nonlinear optical phenomena can be involved, which can be placed into two classes: parametric and non-parametric processes [1,2,3,4]. Second-harmonic generation (SHG), sum-frequency generation (SFG), difference-frequency generation (DFG), third-harmonic generation (THG), and four-wave mixing (FWM) belong to the former class, while stimulated Raman scattering (SRS) and stimulated Brillouin scattering (SBS) belong to the latter one. Today, due to the diffraction limit, high-density photonics components do not have the ability to confine light down to the nanoscale dimension, nor to interact with light in an efficient way. Therefore, efficient light conversion, obtained through nonlinear optical processes at the nanoscale, is still a challenge [5,6,7,8,9,10].

The SRS phenomenon occurs in the presence of high energy transfer from a high-power pump beam to a probe beam (copropagating or counterpropagating) [11,12,13,14]. This energy exchange occurs when the frequency difference between the pump and the Stokes laser beams matches a given molecular vibrational frequency of the sample under test; the SRS effect occurs in the form of the gain of Stokes beam power (stimulated Raman gain, SRG) and the loss of pump beam power (stimulated Raman loss, SRL). SRS depends on the pump intensity and on the gain coefficient, which is proportional to the spontaneous Raman scattering cross section and inversely proportional to the linewidth of the corresponding Raman line. Because of its coherent nature, the molecular bonds oscillate in the phase and interfere constructively inside the focus area of the laser beam. As a consequence, the SRS signal, which is orders of magnitude bigger than that of spontaneous Raman scattering, is generated. Due to its Raman-shifted output, SRS is a workable method for generating coherent radiation at new frequencies. SRS permits, in principle, the amplification of wavelengths in a wide interval, from the ultraviolet to the infrared ones [15,16,17,18]. Since the Raman frequency of a medium is usually fixed, the tunability can be achieved by using a tunable pump laser.

Important accomplishments related to integrated laser sources based on SRS have been achieved in the last two decades in the fields of photonics [19,20], microphotonics [21,22,23], and nanophotonics [24,25,26]. In the investigation of SRS at the nanoscale, how it often happens for nonlinear optics phenomena and fundamental and applicative issues are two faces of the same coin. Concerning the fundamental ones, there have been several investigations, both experimental [27,28,29,30,31] and theoretical [32,33,34,35,36,37,38] ones, but the “question is still open,” while from an applicative point of view, there are some important perspectives to realizing micro-/nano-sources with improved performances. Although a general theory on the relationship between nanostructuring and Raman gain has not been established, we expect that when the particle dimensions are of a few nanometers, the phonon confinement effect plays a significant role; therefore, SRS enhancement should be attributed to the quantum confinement effect, and the gain of nanomaterials should be different from bulk and related to the intrinsic properties of materials [32,33,34,35,36,37,38].

Among the nonlinear nanostructured materials, which are generally formed by an active material (rare-earth ions, quantum dots, metal clusters or nanoparticles, and organic and carbon-related materials) uniformly dispersed in a host matrix, inorganic glasses and transparent glass-ceramics (TGCs) play a key role due to their chemical stability and their linear and nonlinear optical features, which can be finely tuned by changing the chemical composition and applying suitable heat treatments [39,40,41,42]. In a glassy matrix, structural transformation at the nanoscale can occur by phase separation when it takes place as a binodal mechanism. In this case, the development of the new liquid phase occurs by nucleation and the subsequent growth of nuclei into droplets, whose size may be controlled by a proper heat treatment [43]. Depending on the composition of the initial glass and the heat treatment temperature, this process produces either two amorphous phases (amorphous nanostructuring) or the growth of a crystalline phase uniformly dispersed in the amorphous phase (crystalline nanostructuring), thus producing TGCs [44,45,46].

The optical efficiency of TGCs is related to scattering phenomena originated by nanocrystals, which are randomly distributed in the glassy matrix, acting as scattering centers [42,46]. Scattering is influenced by the difference in refractive indexes between the amorphous and crystalline phases, by the size and distribution of crystals in the glassy matrix [42], and by the crystallization mechanism in the active phase [42,44,45,46]. The high anisotropy of a non-centrosymmetric crystal usually induces surface nucleation, making bulk nucleation a precondition to obtain a transparent nanostructure with nonlinear optical (NLO) properties [46]. Therefore, TGC were obtained using either bulk nucleating phases, such as NaNbO_3_ [42], LiNbO_3_ [47], Ba_2_NaNb_5_O_15_ [46], or using suitable nucleating agents to promote bulk nucleation [42]. It is worth noting that transparent nanostructured glass-ceramics obtained by a glass with molar composition 8BaO·15Na_2_O·27Nb_2_O_5_·50SiO_2_ (BaNaNS) by means of a bulk nucleation mechanism and suitable heat treatments exhibit a distinct diffuse second harmonic activity, which is ascribed to crystalline nanostructuring [46].

Today, in order to satisfy the increasing telecommunication demands [48,49], the usefulness of existing Er-doped fiber amplifiers is reduced, leaving Raman gain as the main mechanism for future amplification. In addition, pure silica- and germanium-doped silica fibers, which are currently utilized as Raman gain media in telecommunications, have quite low Raman gain coefficients and a limited usable spectral bandwidth. Therefore, the investigation of new materials possessing both large Raman gain coefficients and spectral bandwidth is becoming necessary. Although tellurite- [50,51] and chalcogenide [52,53]-based glasses combine large scattering intensity and bandwidth, giving very high Raman gains that are up to 40 times larger than that of SiO_2_, their fabrication demands caution. In our previous papers, SRS investigations in nanometrically heterogeneous glasses in the K_2_O-Nb_2_O_5_-SiO_2_ (KNS) system have been reported [54,55], demonstrating an enhancement of Raman gain with respect to silica and a broadening of Raman gain spectra.

Herein, a deep investigation of SRS in TGCs based on bulk nucleating phase Ba_2_NaNb_5_O_15_ is reported. The aim is to demonstrate that SRS can be finely tuned by controlling the structural transformation at the nanoscale, as probably occurred for the previously investigated SHG [46], excluding any contributions deriving from microstructural changes. To achieve this, different and complementary techniques were used such as differential thermal analysis (DTA), transmission electron microscopy (TEM), scanning electron microscopy (SEM), quantitative chemical elemental analyses (EDS), X-ray diffraction (XRD), optical microscopy (polarized light), ellipsometry, and Raman spectroscopy.

## 2. Materials and Methods

### 2.1. Preparation of Samples and Thermal Analysis

The glass with molar composition, 8BaO·15Na_2_O·27Nb_2_O_5_·50SiO_2_ (BaNaNS), was synthesized using reagent grade NaNO_3_, BaCO_3_, Nb_2_O_5_, and SiO_2_ according to the procedure reported elsewhere [56]. Optically transparent, brown-colored, amorphous samples were obtained, the majority of which did not contain crystalline inclusions. Samples containing dendritic crystalline inclusions were obtained too.

The devitrification behavior of the glass was studied by differential thermal analysis (DTA) using a TA thermoanalyzer (SDT Q600), with Al_2_O_3_ as reference material. DTA curves were recorded in air on bulk specimens of about 20 mg at the heating rate of 10 K min^−1^. The accuracy of detected temperatures in DTA curves was ±1 K. Heat treatments were performed in the DTA apparatus to eliminate temperature gradients.

### 2.2. Structural Characterization

#### 2.2.1. XRD and TEM

XRD profiles were recorded using a Philips automatic diffractometer using Ni-filtered CuKα radiation (λ = 0.15418 nm). The degree of crystallinity X_c_ was evaluated considering the subtended areas in the XRD profiles of the sample (As) and of the amorphous phase (Aa) using the formula X_c_ = 100 (As − Aa)/As [57].

All the samples for XRD measurements were heat treated in the DTA furnace by quenching them directly after that either a DTA peak occurred or at the end of the heating program.

TEM images were acquired to investigate the morphology of Ba_2_NaNb_5_O_15_ crystals using a FEI TECNAI G2 200 kV microscope. Approximately 5 µL of a suspension containing the powdered sample in methanol was placed on a carbon-coated copper grid and allowed to air dry before imaging.

#### 2.2.2. Scanning Electron Microscopy (SEM) and EDS

Textural, morphological, and quantitative chemical analyses were performed using a field emission scanning electron microscope (FE-SEM) (Merlin VP compact with Zeiss Gemini camera) coupled with an energy dispersive X-ray spectroscopy (EDS) (Oxford Instruments Microanalysis). The setup operated with a primary beam voltage of 15 kV, a working distance of 8.5 mm, and opening at 60 µm with high current. Data were processed with the INCA software, taking advantage of the matrix correction scheme developed by Pouchou and Pichoir [58]. The following natural and synthetic standards were used for calibration: anorthoclase for Si and Na, benitoite for Ba, and NbOPO_4_ for Nb. One glass fragment for each sample was mounted in epoxy resin, carefully polished, washed in ultrasonic bath, and dried in air. Afterwards, the samples were spatter-coated with a thin carbon film for EDS analyses. Twenty analytical points were collected for each glass samples, and there were five analytical points for 4 microlites in each sample.

#### 2.2.3. Linear and Nonlinear Optical Characterization

##### Optical Microscopy

In this study, the acquisitions were made in polarized transmitted light using an Zeiss Axio Imager Am1 microscope. The camera used was AxioCam ICc,5 while the image management software was Zeiss Axiovision 4.9 with the active module Automeasure.

##### Ellipsometric Characterization

Ellipsometric measurements were carried out using a Jobin Yvon UVISEL-NIR phase modulated spectroscopic ellipsometer apparatus according to a previously described procedure in our papers [54,55].

##### Raman Characterization

A confocal Raman microscope (Jasco, NRS-3100) was used to obtain Raman spectra. The 514 nm line of an air-cooled Ar^+^ laser (Melles Griot, 35 LAP 431220) at 125 mW was injected into an integrated Olympus microscope and focused on the sample with a spot size of approximately 3 μm at 20× magnification with a final power of 2.5 mW. A holographic notch filter was used to reject the excitation laser line. The Raman backscattering was collected at 180° using a 0.1 mm slit and a diffraction lattice of 1200 grooves/mm, corresponding to an average spectral resolution of 8 cm^−1^. It took 60 s to collect a complete data set using a Peltier-cooled 1024 × 128 pixel CCD photon detector (Andor DU401BVI). Wavelength calibration was performed by using polystyrene as a standard. Raman microscopy measurements on BaNaNS samples were performed on the smoothest surface, and spectra were recorded at three distinct locations of the sample to test for homogeneity and at three different depths to test for reproducibility of the Raman intensity.

As the intensity of the Raman active modes depends on the temperature and on the frequency of the vibrational modes, the measured Stokes Raman intensity *I*(*ω*) was reduced according to the following relation:

(1) $$R\left(\omega \right)=\frac{\omega }{N\left(\omega ,T\right)+1}\frac{1}{{\left(\omega_{0}-\omega \right)}^{4}}I\left(\omega \right)\;$$

where *ω* is the Stokes Raman shift, *ω*_0_ is the laser excitation frequency (both in cm^−1^ units), *N*(*ω*,*T*) is the Bose–Einstein mean occupation number, and *T* is the temperature. Afterwards, to correctly compare the Raman spectra of BaNaNS glasses with the silica glass standard, the data must also be corrected for the differences in reflection and angle of collection [54,55]. Reduced and corrected Raman spectra were fitted to several Gaussian components. The Raman gain spectrum is related to the spectral and differential Raman cross section by means of the following equation [54,55]:

(2) $$g\left(\omega \right)=\frac{\lambda_{S}^{3}}{c^{2}hn^{2}}{\left(\frac{\partial^{2}\sigma }{\partial \Omega \partial \omega }\right)}_{0}$$

where *c* is the velocity of light in vacuum, *n* is the refraction index at the excitation wavelength, *h* is Planck’s constant, and *λ_S_* is the Stokes wavelength (in meters) defined as:

(3) $$\lambda_{S}=\frac{10^{7}}{\left(\omega_{0}-\omega \right)}$$

The Raman cross section at *T* = 0 K (i.e., corrected for the thermal population factor) is given by:

(4) $${\left(\frac{\partial^{2}\sigma }{\partial \Omega \partial \omega }\right)}_{0}=\frac{1}{1+N\left(\omega ,T\right)}\left(\frac{\partial^{2}\sigma }{\partial \Omega \partial \omega }\right)$$

where $\left(\frac{\partial^{2}\sigma }{\partial \Omega \partial \omega }\right)$ is the measured Raman cross section at temperature *T*.

## 3. Results and Discussion

Because this study aimed to clarify the correlations between structural transformations of the glassy matrix and NLO properties, all the results were collected on glass specimens that were free of defects. Nevertheless, to focus on the different role of the distinct possible phases present in the glass (bulk, crystalline microdomains, and nanodomains) in modulating its NLO properties, we also selected and examined glass specimens with crystalline inclusions (microdomains) [59]. Optical microscopy was used to identify the presence and spatial distribution of microdomains and to assist with the elemental analysis of the subsequent SEM-EDS investigation of both the bulk and microdomain phases.

It should be emphasized that the XRD profiles of samples containing crystalline inclusions do not show interference XRD peaks, resulting in an identical XRD pattern to that of selected glass sample without crystalline inclusions. This result suggests that the fraction of crystalline inclusions can be considered as negligible with respect to the amorphous phase.

TGCs based on Ba_2_NaNb_5_O_15_ nanocrystals were obtained by heat treatments performed on BaNaNS glass specimens at temperatures similar to the first DTA exothermic peak: 30 min at the extrapolated onset (700 °C) and/or at the end temperature (745 °C) [46]. It was shown that this glass is stable (easily forming) and devitrified, giving only the non-centrosymmetric Ba_2_NaNb_5_O_15_ phase due to a bulk nucleation mechanism [46]. It should be underlined that the crystallization from a glass of a polar phase characterized by a large amount of anisotropy generally occurs due to a surface nucleation mechanism. The latter one does not allow the control of transformations occurring at the nanoscale during the transformation of glass into crystals obtained by tuning the temperature. Therefore, Ba_2_NaNb_5_O_15_ bulk nucleation from BaNaNS glass is a precondition to tune the glass nanostructure, and in turn, the NLO properties of the transparent glass-ceramic.

**Figure 1:**
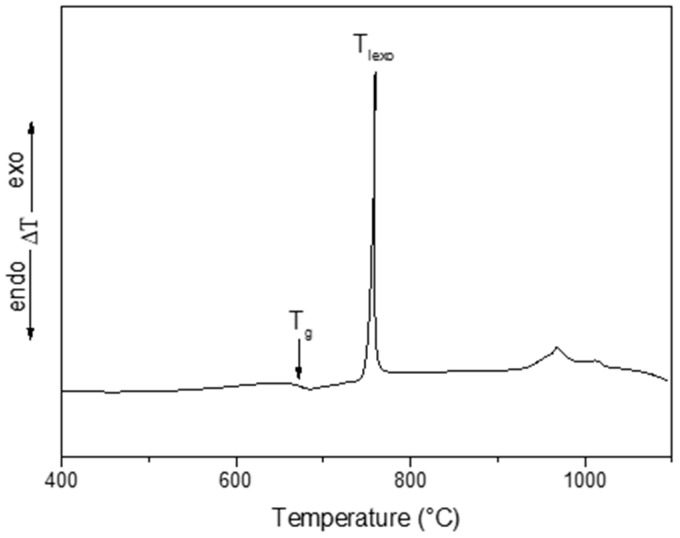
DTA curve of the bulk initial BaNaNS glass recorded in air at 10 K·min^−1^.

The DTA curve of the BaNaNS glass is displayed in Figure 1. The slope change in the baseline can be related to the glass transition (second order transition). Here, the inflection point of this slope change is indicated as the glass transition temperature, T_g_, at 670 °C (see Figure 1).

It was shown that at the temperature of the first exothermic DTA peak (T_Iexo_ = 758 °C), crystallization of Ba_2_NaNb_5_O_15_ occurs [46]. This phase belongs to the tetragonal tungsten bronze (TTB) family, which is a set of non-centrosymmetric crystals showing good NLO properties [60]. TTB compounds have a general formula of (A1)_2_·(A2)_4_·(C1)_4_ (B1)_2_·(B2)_8_·O_30_, where the five kinds of sites are available to be filled by different atoms. They allow the formation of many isomorphous crystals, showing NLO properties in a wide compositional range [61,62]. In our case, square A1 sites are occupied by Na, pentagonal A2 sites are occupied by Ba, and B1 and B2 octahedral sites are occupied by Nb, whereas the C sites are empty. It was shown that the Ba_2_NaNb_5_O_15_ phase crystallizes due to a bulk nucleation mechanism [46]. As known, in glass, the maximum of nucleation rate occurs at T_g_; therefore, to increase the number of crystalline nuclei, isothermal nucleation heat treatments were performed for different times, T_g_.

Owing to high number of crystalline nuclei, the DTA crystallization peak of nucleated samples shifts towards lower temperatures. The largest shift (T_Iexo nu._ = 730 °C), which was observed for the 6h heat treatment, produces the highest number of nuclei. This down shift is a consequence of the formation of many crystalline nuclei uniformly dispersed in the glassy matrix. They facilitate the growth of a very high number Ba_2_NaNb_5_O_15_ nanocrystals, preserving the full transparency of the sample [46]. Based on these data, TGCs were obtained by the following heating programs performed on samples that were heat treated 6 h at T_g_ (nucleated samples). Program (1): heated at 10 K·min^−1^ up to the temperature of the first DTA exothermic peak (T_Iexo nu._ = 730 °C); program (2): heated at 10 K·min^−1^ up to the end temperature of the first DTA exothermic peak (T_end nu._ = 745 °C) and held at this temperature for 30 min; program (3) heated at 10 K·min^−1^ up to the extrapolated onset temperature of the first DTA exothermic peak (T_ext nu._ = 700 °C).

As later described, the analysis of the Raman spectra of samples subjected to the above thermal programs evidence that program (3) gives rise to an intermediate stage between the initial glass and the fully nanostructured samples. This indicates that at this stage, the nanocrystallization process is not yet complete, whereas its completion takes place only after the programs 1 and 2, where the highest Raman gain values were obtained. Consequently, in the following part, we focus our attention only on the glass subjected to heating programs (1) and (2).

**Figure 2:**
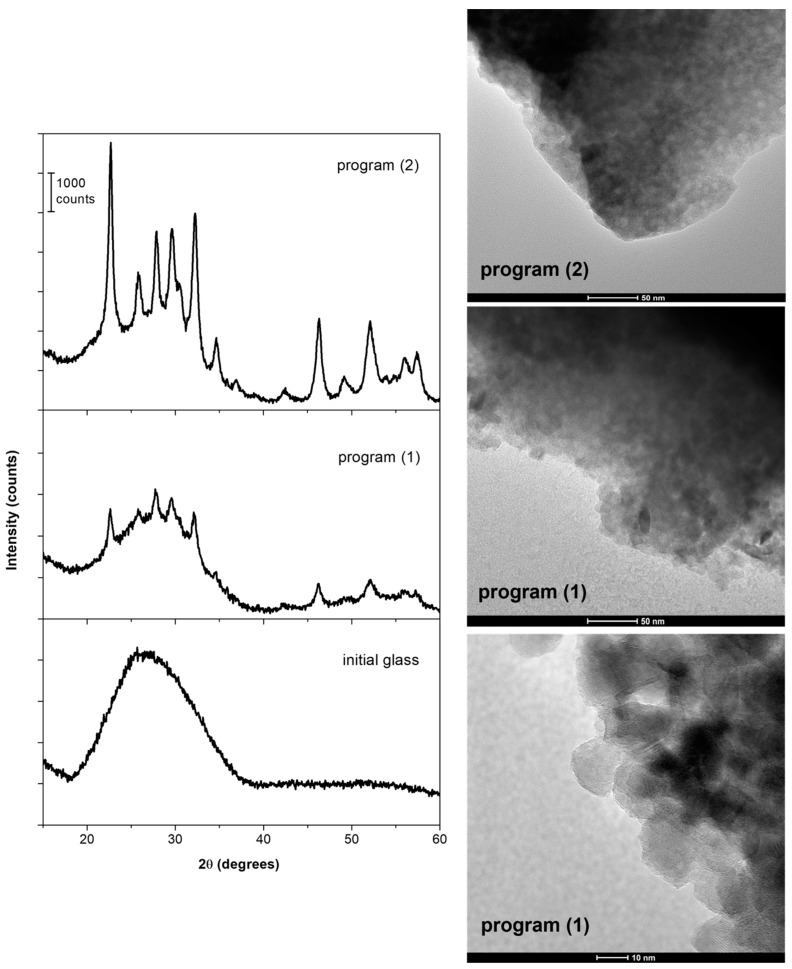
Left column: XRD patterns of initial, nucleated BaNaNS glass subjected to thermal program (1) and to program (2) (from the bottom). Right column: TEM images of nucleated BaNaNS glass subjected to thermal programs 1 and 2.

In Figure 2, XRD patterns of the initial BaNaNS glass and of nucleated BaNaNS glasses subjected to program (1) and to program (2) are shown. The initial glass is amorphous, and from its XRD pattern, the fact that a very small amount of crystalline inclusion formed during the quenching process cannot be ruled out. The XRD pattern of nucleated BaNaNS glass subjected to thermal program (1) shows the presence of low-intensity diffraction peaks and a non-negligible amorphous halo, indicating that the thermal treatment induces the partial crystallization (degree of crystallinity~30%) of Ba_2_NaNb_5_O_15_ structures (ICDD 86-0739). The sample subjected to program (2) presents an XRD diffraction pattern with well-defined diffraction peaks that are related with the higher degree of crystallinity (~50%) observed during thermal treatment 2. The crystallites size, which is evaluated through the Scherrer equation (considering the peak at 2θ = 22.6°), is about ~15 nm for both samples indicating that the different thermal treatments induce a different degree of crystallinity, but do not substantially affect the crystal size.

The TEM images collected of samples prepared with both thermal programs (1) and (2) show a uniform distribution of nanocrystals in the residual glassy matrix (Figure 2). The nanocrystals size is the same (10–15 nm), as evaluated from the XRD diffraction profiles.

Ba_2_NaNb_5_O_15_ nanocrystals are clearly visible in the TEM image of the sample obtained by the thermal program (2) collected at high magnification (Figure 2), where both the crystal size (10–15 nm) and the crystal lattice can be observed.

As said before, the as-quenched BaNaNS glass samples were carefully inspected to select the specimen that was free of any crystallite for further study. However, some glass samples containing some crystallites formed during the melt quenching were also investigated by optical microscopy and SEM to understand their nature and genesis. It is worth remembering that even when crystalline inclusions are present in the as-quenched BaNaNS glass sample, its XRD pattern appear to be fully amorphous (identical to that of the sample without inclusion showed in Figure 2), indicating their very small amount. Before characterizing the crystalline inclusions, it must be remembered that, as is known, during the cooling of the melt, crystals of the thermodynamically more stable phase are formed. On the other hand, when a glass is heated up to the crystallization temperature, the kinetically favored crystalline phase grows, namely, the phase that grows faster, giving a metastable crystalline phase that converts into a stable one only at a higher temperature. This transformation, being a solid-state one, occurs at a much higher temperature.

**Figure 3:**
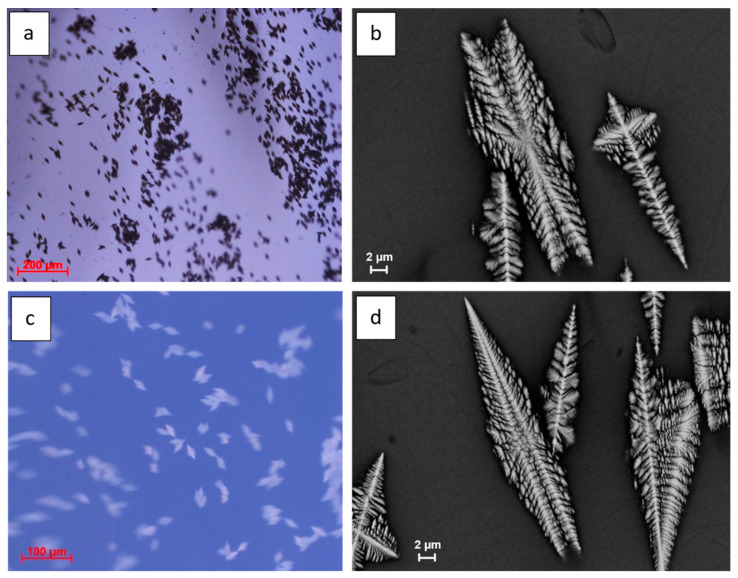
(**a**) Polarized light image and (**b**) SEM image (magnification 2000×) of subparallel layers of microinclusions in a sample of the initial BaNaNS glass. (**c**) Crossed polarized light image with converging lens and (**d**) SEM image (magnification 2000×) of a random multidomain nanostructured BaNaNS glass (heat program (2)). In (**c**), the glass matrix is clearly blue, whereas the solid inclusions are white.

The investigation of BaNaNS glasses by polarized light microscopy showed, in some samples, the presence of crystalline inclusions with variable dimensions (10–20 μm) grouped in subparallel layers (Figure 3a). The inclusions are characterized by a specific morphology such as biterminated spears. Higher magnification SEM images show their typical dendritic shape (Figure 3b). The quantitative chemical data (EDS) show different composition for glass and microlites. For the glass matrix, the chemical average composition is: BaO = 9.71 (±0.2) wt%, Na_2_O = 7.30 wt% (±0.1), Nb_2_O_5_ = 58.57 wt% (±0.5), SiO_2_ = 23.51 wt% (±0.2). For microlites, the chemical average composition is: BaO = 20.19 wt% (±0.7), Na_2_O = 4.60 wt% (±0.2), Nb_2_O_5_ = 61.22 wt% (±1.5), SiO_2_ = 13.05 wt% (±2.0).

**Figure 4:**
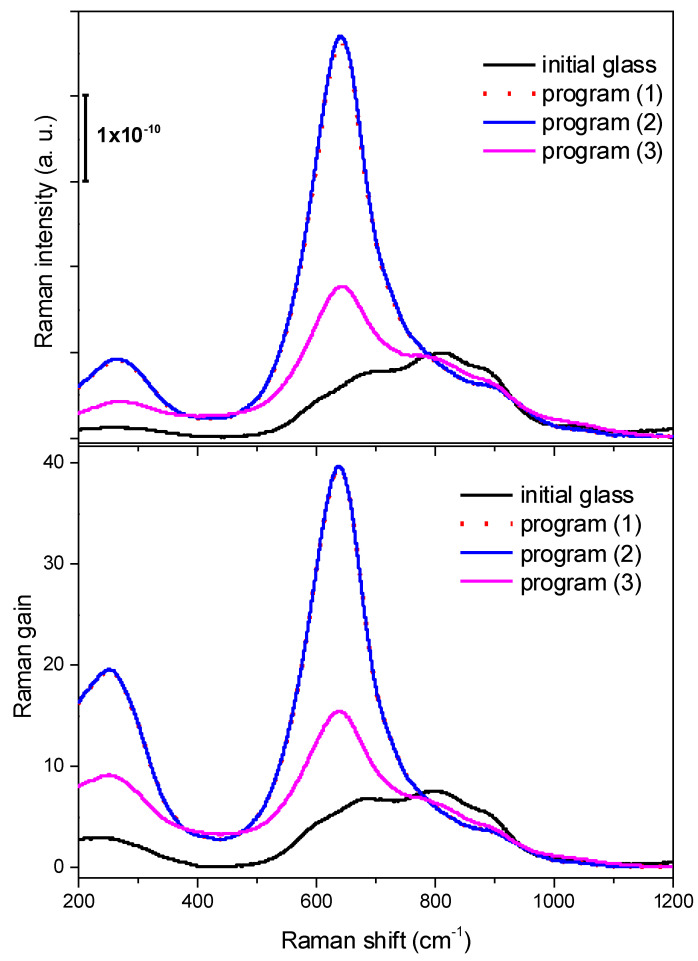
Reduced Raman spectra (**up**) and Raman gain (**down**) of the initial BaNaNS glass and nucleated samples subjected to different thermal treatments.

The BaNaNS initial glass, as well as the nucleated glass samples subjected to heating programs (1), (2), and (3), were fully characterized by spectroscopic ellipsometry and Raman spectroscopy. Refractive indices (*n*) of all these samples measured at 514 nm are reported in Table 1. The reduced Raman spectra and corrected Raman spectra displayed in Figure 4 were fitted to several Gaussian components, and the results are listed in Table 1.

Raman spectroscopy is particularly sensitive to structural modifications of highly polarizable bonds, such as Nb-O. Previous works on Nb-Si-metal cations (such as Na^+^, K^+^) by the authors allowed them to identify several possible coordination of Nb-O species, mostly involving octahedral and tetrahedral geometry, which were more or less distorted [54,55]. In our system, where the metal cations are Ba^2+^ and Na^+^, Raman spectra clearly reveal how Nb-O coordination undergoes significant structural modifications during thermal treatments, going from the initial glass (black line in Figure 4) to the nucleated samples that were kept at T_g_ 670 °C for 6h, and then subjected to heating program (3) (magenta trace in Figure 4) and heating programs (1) and (2) (red, dotted line and blue line in Figure 4, respectively). The thermal treatments considerably modify both the wavenumbers and the intensity of the Nb-O related Raman bands, suggesting a significant structural modification of the Nb-O network, which is interpreted as ongoing nanostructuring [54,55].

The Raman spectra of these glasses are typically composed of a low-frequency region (200–400 cm^−1^) assigned to bending modes of Nb−O−Nb bonds and a higher frequency region (500−900 cm^−1^) related to vibrational modes of NbO_6_ octahedra characterized by a different distortion degree.

Typically, low-distorted NbO_6_ octahedra (i.e., octahedra with no nonbridging oxygens) and highly distorted NbO_6_ octahedra (i.e., octahedra with nonbridging oxygens) occur at lower and higher wavenumbers, respectively [54,55]. The initial BaNaNS glass is characterized by broad components with features at about 680, 810, and 890 cm^−1^ (related to NbO_6_ octahedra with different distortion degree), whereas the thermally treated nucleated samples are dominated by bands in the range of 500–700 cm^−1^, exhibiting a narrow and strong band at about 640 cm^−1^. This feature is associated with structured nanodomains that foster the formation of NbO_6_-ordered octahedra clusters, shifting to lower frequency the vibrational modes of Nb−O bonds.

Concerning the Raman gain, it is worth noting that for a number of materials, for example silicon, a direct measurement is possible. However, when the Raman gain is not so high and the length of the sample is small, an indirect measurement must be utilized. Usually, this happens, for example for glasses; in this case, the Raman gain is estimated by a numerical procedure starting from experimental data provided by spontaneous Raman scattering [54,55].

The Raman gain spectra of investigated samples normalized to the one of silica were calculated and are reported in Figure 4.

For the initial glass, the value of Raman gain peak was evaluated to be 7.5 times that of silica. It increases up to 15 and 40 for the samples obtained from heating program (3) and the (1) and (2), respectively, according to the above structural transformations. Noticeable modifications in the Raman spectra and a significant enhancement of Raman gain (up to 40 times higher) of both the BaNaNS glasses and annealed samples with respect to initial one and to SiO_2_ glass due to nanostructural transformation that occurred during the thermal treatment are demonstrated.

The data collected in this study concern the nanostructured systems obtained when BaNaNS glass was subjecting to suitable heat treatments. These data clearly show that the structural transformations occurring at the nanoscale are responsible for the observed SRS effects. In particular, the bulk nucleation mechanism of the Ba_2_NaNb_5_O_15_ phase is the key factor that controls the nanostructure. The low level of overlapping between the nucleation and crystals growth curves facilitates the growth of a very high number of nanocrystals, subjecting the nucleated samples to suitable treatments in a temperature range, where crystallization occurs [45]. We have previously demonstrated that the 6 h heat treatment at T_g_ does not change the glass microstructure, but it produces a bulk nucleation of the Ba_2_NaNb_5_O_15_ phase [46]. Therefore, heating up to 730 °C (T_Iexo nu._) or the heat treatment for 30 min at 745 °C (T_end nu._) of the nucleated samples produces only a slight increase in the crystallization degree, going from 30 to 50%, but it does not significantly change the size of the nanocrystals, which are about 15 nm as attested by TEM and XRD analyses (see Figure 2). On the contrary, any NLO effect was detected for samples containing microdomains, indicating that the microstructure has a negligible role on the SRS effect.

## 4. Conclusions

In this paper, TGCs based on Ba_2_NaNb_5_O_15_ nanocrystals were obtained by proper thermal treatments of nucleated BaNaNS glass samples (heating either up to the temperature of the first DTA exothermic peak or up to the end temperature of the first DTA exothermic peak and holding this temperature for 30 min). These TGCs are formed by Ba_2_NaNb_5_O_15_ nanocrystals of the same size (10–15 nm), but different crystallinity degrees (30 and 50%). These crystalline nanodomains are richer in niobium with respect to the residual glass matrix and give rise to TGCs with SRS activities.

The Ba_2_NaNb_5_O_15_ nanocrystals randomly dispersed in the glassy matrix play a fundamental role in determining the SRS effect as well as occurred for SHG activity [46], definitively demonstrating that the NLO properties both in the second and at the third order are ruled by the structural transformation occurring at the nanoscale. Based on these findings and considering the previously reported SHG activity, the proposed materials appear to be promising for future applications of photonics nonlinear conversion.

## Figures and Tables

**Table 1:** Raman and refractive properties of a silica glass and of the initial BaNaNS glass and nucleated samples subjected to different thermal treatments.

	Refractive Index	Peak Position (cm^−1^)	FWHM (cm^−1^)	Sigma/Sigma_SiO_2__	I/I_SiO_2__	g/g_SiO_2__
SiO_2_ glass	1.461	450	190	1	1	1
Initial BaNaNS glass	1.983	A = 685 B = 800 C = 886	110 60 70	71 32 41	52 43 46	7.5
Nucleated BaNaNS glass subjected to thermal program (1)	2.014	A = 640 B = 810 C = 915	80 105 100	292 62 19	292 47 15	40
Nucleated BaNaNS glass subjected to thermal program (2)	2.025	A = 640 B = 810 C = 915	80 105 100	292 62 19	292 47 15	40
Nucleated BaNaNS glass subjected to thermal program (3)	1.989	A = 640 B = 808	70 160	91 119	104 60	15

## Data Availability

Data is contained within the article.
